# An Introduction to Terminology and Methodology of Chemical Synergy—Perspectives from Across Disciplines

**DOI:** 10.3389/fphar.2017.00158

**Published:** 2017-04-20

**Authors:** Kyle R. Roell, David M. Reif, Alison A. Motsinger-Reif

**Affiliations:** ^1^Bioinformatics Research Center, North Carolina State UniversityRaleigh, NC, USA; ^2^Department of Biological Sciences, North Carolina State UniversityRaleigh, NC, USA; ^3^Department of Statistics, North Carolina State UniversityRaleigh, NC, USA

**Keywords:** synergy, mixtures, non-additive, interactions, drug combinations

## Abstract

The idea of synergistic interactions between drugs and chemicals has been an important issue in the biomedical world for over a century. As complex diseases, especially cancer, are being treated with various drug cocktails, understanding the interactions among these drugs is increasingly vital to ensuring successful treatment regimens. However, the idea of synergy is not limited to only the biomedical realm and these ideas have developed across many different disciplines, as well. In this review, we first discuss the various terminology surrounding the idea of synergy, providing a comprehensive list of terms defined across numerous disciplines. We then review the most common methodology for detection and quantification of synergy, including the two most prominent reference models for describing additive interactions: Loewe Additivity and Bliss Independence. We also discuss advantages and limitations to each method, with a focus on the Chou-Talalay Combination Index method. Finally, we describe how methods development and terminology have developed among disciplines outside of biomedicine and pharmacology, to synthesize the literature for readers.

## Introduction

The idea that 1 + 1 = 2 is not novel and virtually agreed upon and understood throughout various academic disciplines and even different cultures around the world. So, it's very counterintuitive to say 1 + 0 = 2, 0 + 0 = 1, or even 1 + 1 = 0. However, that paradigm is a very basic analogy for understanding synergist interactions, or synergy. Synergy is commonly defined as the effect of two or more agents working in combination that is greater than the expected additive effect of said agents (Greco et al., 1996). Now, going back to the 1 + 0 = 2 analogy (Berthoud, 2013; Geary, 2013), it can be asserted that there is a synergistic interaction occurring. Unfortunately, in practice, it is far from that simple to quantify such interactions.

Synergy is most often defined in relation to the realms of pharmacology and medicine. This is because many diseases require treatment that consists of “cocktails,” or mixtures of various drugs taken at once. This is particularly true in cancer treatment. This potentially allows for maximizing the therapeutic effect while minimizing the adverse effect, or side effects, upon being treated with a given drug regimen (Greco et al., 1996; Foucquier and Guedj, 2015). If two drugs act synergistically, lower doses of each drug could potentially be used which could allow for less adverse effects while still provided the desired outcome, such as cancer cell death.

There are a number of examples of clinical synergy in the area of cancer treatment, often established between established chemotherapy agents and agents representing other classes. Herceptin has been shown to synergistically interact with both doxorubicin and paclitaxel, though cardiotoxicity risk is increased in combination therapy (Pegram et al., 2000). Another example of this has been shown to between anticancer drugs edatrexate and cisplatin as well as methotrexate and cisplatin, resulting in the ability to use lower doses of each drug in combination while maintaining the same cell death levels as when using higher doses of these drugs individually (Chou et al., 1993). The addition of a complement-fixing monoclonal antibody, rituximab, to cyclophosphamide, hydroxydaunomycin, Oncovin, and prednisone (CHOP) therapy in non-Hodgkin's lymphoma increases the overall response without increasing toxicities (Weiner, 2010). Finally, the long-established combination of vincristine and prednisone is highly potent blood born tumors, with the addition of the anthracyline crucial for cure, not just remission (Weiner, 2010).

The majority of this review will discuss synergy in relation to the domains of pharmacology and biomedicine, however there are a range of applications of synergy that can be seen among various non-clinical fields. Synergistic combinations have been observed in chemical mixtures more generally (Carpenter et al., 2002), often evaluated in the realm of toxicology and environmental health. For example, Laetz et al. describe the effects of synergistic neurotoxicity in salmon when exposed to various chemical mixture of heavily used insecticides (Laetz et al., 2009). While the overall properties of synergistic interactions are the same across fields, as often happens in the scientific literature, separate methods and corresponding terminology emerge across disciplines. The use of different “jargon” is a challenge for interdisciplinary research, as it limits cross-talk across methods developed in different domain applications. In this review, we will unify the various terms that have developed across disciplines in a comprehensive list, to enable researchers to more readily synthesize the diverse literature. We will also review the statistical and computational approaches used to quantify synergistic interactions, and discuss their relative advantages and disadvantages. Throughout this review, empirical examples of synergy using the described methods and among disciplines can be found in the referenced reviews papers and experimental articles.

## Terminology

As models and methods for detecting synergy among interactions have developed over the past century, terminology used across the various fields and within these methods has not always been consistent. This inconsistency can even be seen within the field of biomedicine, one of the most common fields to apply methods for synergy detection. This results in potential confusion when trying to discern which methods best suit a given situation. Here, we offer a comprehensive list of some common terms in defining synergy and their respective equivalencies to help alleviate any potential conflicts and inconsistencies in defining and quantifying synergy.

The first definition necessary when regarding synergy is the concept of additive effects. This has also been referred to as noninteraction, and inertism (Greco et al., 1995). An additive effect is generally considered as the baseline effect for synergy detection methods. It is the effect that is theoretically expected from the combination of multiple drugs when synergy is not present. Although seemingly simple from its name, the idea of simply adding two, or more, effects together do not accurately reflect what happens realistically. A quick, simple example to show this involves two separate drugs A and B that both exhibit 60% cell death at a saturating dose, for example. Would it be possible to simply add these effects together to get a combined effect of 120% cell death; no, that is not realistically possible (Chou, 2010). The problem of mathematically defining additivity has been the center of controversy among leading researchers of this topic for the last century. However, there are two models that have prevailed and will further be described in the Reference Models section. Along with these prevailing models, there are two additional terms, Loewe Additivity and Bliss Independence (Greco et al., 1995), that are essentially synonymous with the basic definition of additive but exist for these specific reference models. Loewe Additivity has also been referenced as dose additivity and Concentration Addition (Cedergreen, 2014). Additionally, Bliss Independence is sometimes referred to as Bliss Additivity (Geary, 2013), Response Multiplication, Response Addition, Effect Addition, and Independent Action (Cedergreen, 2014).

Any (significant) deviation from additivity would be classified as synergy or antagonism. It is often agreed upon that synergy can be defined as a combination effect that is greater than the additive effect expected from good knowledge of the individual drugs. Synergy has also been called superadditivity (Tallarida, 2001), potentiation, augmentation (Berenbaum, 1977), supra-additivity (Geary, 2013). The term coalism is also sometimes used to refer to synergy when neither drug, or none of the drugs in mixtures of more than two chemicals, is effective on its own (Greco et al., 1995). There are also two distinctively termed ideas to describe synergy under the previously mentioned specific models. Those terms are Loewe Synergy and Bliss Synergy; the models from which the terms have been derived will be discussed further in Reference Models.

Antagonism is the opposite of synergy; it occurs when the combined effect of compounds is less than what would be expected. In the biomedical world, it is often considered more of a negative scenario, as many researchers are looking to identify synergistic interactions among compounds for some sort of added therapeutic effect. However, in a toxicological sense, it may be beneficial to have an antagonistic effect in a mixture of chemicals. Antagonism has also been named subadditivity (Tallarida, 2001), infra-additive (Geary, 2013), negative interaction, depotentiation and even negative synergy (Berenbaum, 1977). Synonyms used in discussions of synergy, additivity, and antagonism are summarized in Table 1.

**Table 1:** **Synonyms used in discussions of synergy**.

**Synergy**	**Additivity**	**Antagonism**
Loewe Synergy^*^, Bliss Synergy^*^, superadditivity, supra-additivity, potentiation, augmentation, coalism	Noninteraction, inertism, Bliss Independence^*^, Loewe Additivity^*^	Subadditivity, depotentiation, negative interaction, negative synergy, infra-additive

*Asterisks indicate terms related to specific reference models*.

**Figure 1:**
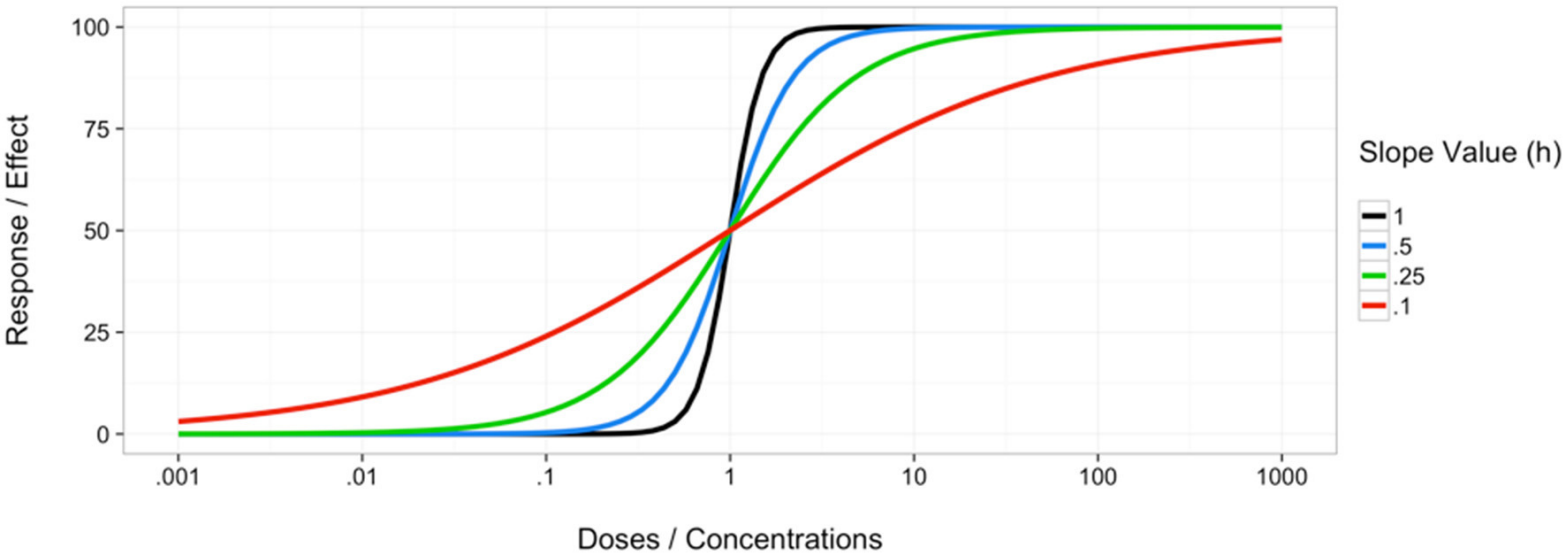
**Example of curves following the Hill equation with different hill slope parameter values**.

Central to some of the methods subsequently discussed is the idea of a dose-response relationship modeled as a curve. This curve is referred to as a dose-response curve, dose-effect curve, concentration-effect curve or concentration-response curve (Chou et al., 1993; Schwartz et al., 2002). These terms are sometimes used interchangeably (Aronson, 2007), but often coincide with the definitions of a dose being the amount of agent administered or experimentally used while a concentration is the measurable amount (typically per some volume of substance) in the experimental system (Nielsen and Friberg, 2013). The experimental system could be an intact organism such as a mouse, or a model system such as lymphoblastoid cell lines, the latter of which has been implicated on various occasions as a viable, high-throughput model for assessing individual drug cytotoxicity (Peters et al., 2011; Brown et al., 2012, 2014; Abdo et al., 2015). A common mathematical model when modeling the dose-response relationship, such as the cytotoxic effect of anticancer agents on cell viability, is the Hill model (Konkoli, 2011; Beam and Motsinger-Reif, 2014). This model was first developed by Hill in 1910 as a model for percent of hemoglobin saturated with oxygen and is now widely used in biological sciences to model various processes, specifically in the form of the Michaelis-Menten equation describing the relationship between enzyme and substrates (Goutelle et al., 2008). This model has also been mathematically rewritten, and even directly referred to, as the sigmoid *E*_*max*_ model, or simply the *E*_*max*_ model or sigmoidal model (Goutelle et al., 2008). The general equation for this model is given in Equation (1), where E is the predicted response of the agent on the system, *E*_0_ is the baseline response for a drug concentration of 0, *E*_*max*_ is the maximum response, *C* is the concentration used for the predicted response *E*, and *EC*_50_ is the concentration for which 50 percent of the maximal response is obtained, and *h* is the hill coefficient of sigmoidicity, also referred to as the slope parameter, which affects the shape of the curve (Goutelle et al., 2008). Examples of this curve and how the slope parameter can shape it are shown in Figure 1. It is also worth noting that while modeling dose-response curves is often a necessary step in many of the synergy detection methods, it is not always a simple task, especially when the curves are nonlinear. However, there have been various approaches to optimizing this procedure, such as an evolutionary algorithm method (EADRM) developed by Beam and Motsinger-Reif (2011).

(1) $$\begin{array}{r} E\;=\;E_{0}\;+\;\frac{\left(E_{max}\;\times \;C^{h}\right)}{\left({{EC}_{50}}^{h}\;+\;C^{h}\right)}\;\left(\text{Hill equation}\right) \end{array}$$

## Reference additivity models

To detect and define synergy, it is first necessary to establish a reference, or “null,” model. This serves as the baseline for quantifying how an interaction between two drugs should occur based on their individual performance, i.e., an interaction that does not exhibit any synergy or antagonism, defined previously as additivity. Deviation from the reference model can then be seen as some sort of synergistic or antagonistic interaction, depending on the direction of the deviation. There have been many attempts at trying to define the best reference model for the general case as well as for specific situations. Upon first glance, it may seem as a rather simple problem, similar to 1 + 1 = 2. However, when considering how drugs interact within the human body, for example, many things are to be considered. Where the drugs are metabolized, the specific target(s), etc., could all contribute to the interaction of the two, or more, drugs used in combination. Unfortunately, a specific reference model cannot take into account every detail of how drugs may interact, and the more general models have prevailed. Throughout the past century, numerous reference models for additive drug interactions have been proposed, however there are two generally accepted models (Greco et al., 1995). Those two models, described as follows, are the Bliss Independence model and the Loewe Additivity model.

### Loewe additivity

The Loewe Additivity model is based on the idea of a “sham mixture,” one where a single drug is mixed with itself. In this type of mixture, there is not expected to be any sort of interaction since a single drug, or various similar drugs, cannot interact with itself, or each other (Loewe, 1926). Thus, the result of a drug combined with itself is called Loewe Additivity. A similar drug would be one with perhaps similar structure and target. This reference model is the basis for many commonly applied methods to detect synergy. As previously stated, it is generally considered one of the two most used reference models, potentially even the most accurate (Greco et al., 1995). However, there are limitations to the model which will be discussed later.

**Figure 2:**
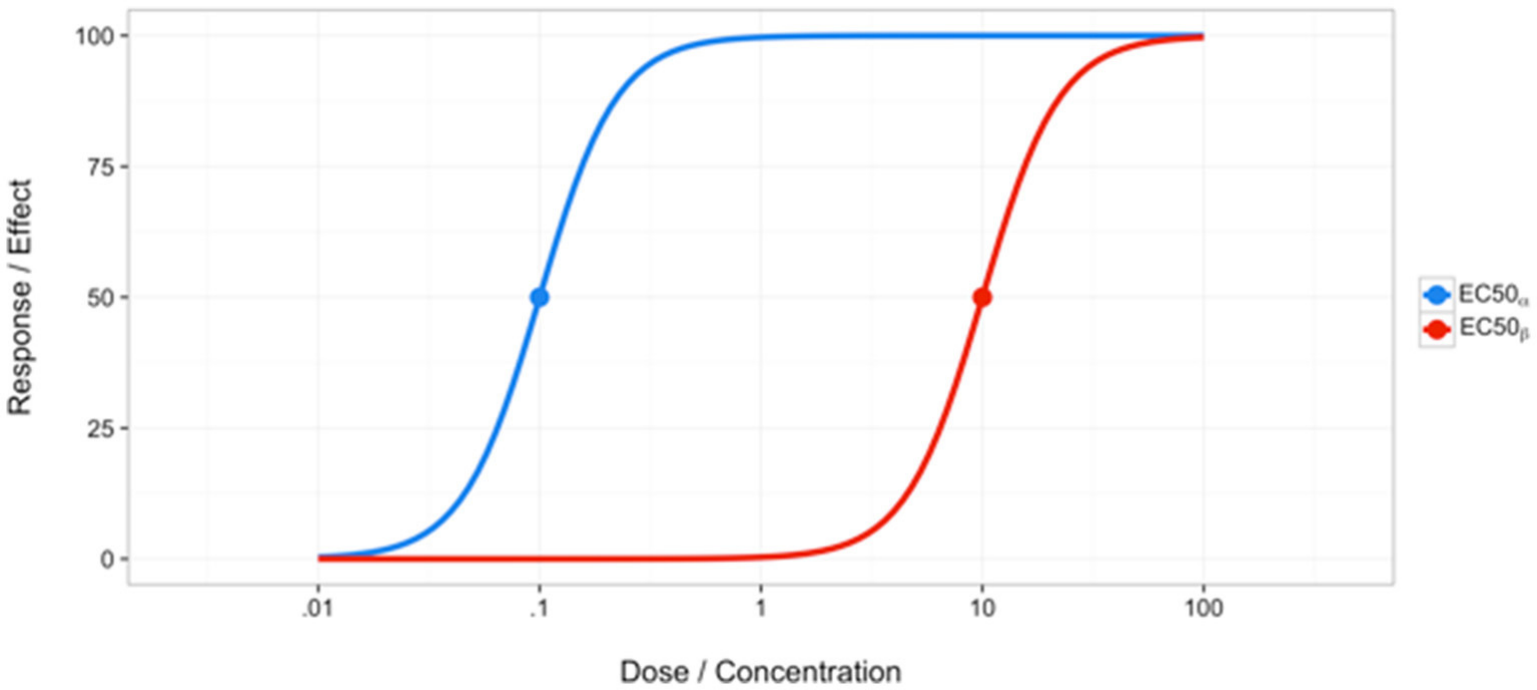
**Example of curves with a constant potency ratio**.

The basic idea of Loewe Additivity assumes a drug cannot interact with itself. Assume two distinct compounds α and β exist and have dose response curves as seen in Figure 2. It is necessary to mention that this model assumes a constant potency ratio, which is the ratio of doses of two individual drugs that give the same effect. Hence, a constant potency ratio is one that maintains some specific ratio $\frac{\text{A}}{\text{B}}$, for example, for all doses (Tallarida, 2012), where *A* and *B* are any doses on curves α and β that achieve the same effect level. It should be noted that in some literature, this has been referred to as a fixed ratio (Hennessey et al., 2010). This can be seen graphically by noticing the parallel structure of the dose response curves for the individual compounds α and β, as shown in Figure 2. A given dose or concentration (ex: EC_50_) that produces a given effect can be measured on either curve. Because the curves have a constant potency ratio, any dose on curve α can be easily translated to its curve β counterpart by taking advantage of this ratio, R = $\frac{A}{B}$. Let us consider measuring some combination of doses, a and b, that produces some effect, F, corresponding to dose A on curve α and B on curve β. To do this, we must first convert the doses to the same curve. As stated, the ratio of doses $\frac{A}{B}$ or $\frac{B}{A}$ permits conversion between curves α and β. Thus, we have:

(2) $$\begin{array}{r} b_{a}\;=\;a\;\times \;\frac{B}{A} \end{array}$$

(3) $$\begin{array}{r} a_{b}\;=\;b\;\times \;\frac{A}{B} \end{array}$$

Where *b*_*a*_ is the dose of compound β needed to achieve the same effect as dose a of compound α, similarly for *a*_*b*_. We can now use to either curve to calculate the effect of

(4) $$\begin{array}{r} F\;=\;\beta \left(b\;+\;b_{a}\right)\;=\;\alpha \left(a\;+\;a_{b}\right) \end{array}$$

(5) $$\begin{array}{r} b\;+\;b_{a}\;=\;B \end{array}$$

(6) $$\begin{array}{r} b\;+\;a\;\times \;\frac{B}{A}\;=\;B \end{array}$$

(7) $$\begin{array}{r} \frac{a}{A}\;+\;\frac{b}{B}=\;1 \end{array}$$

This is the fundamental equation that has come out of the Loewe Additivity reference model. It has been the basis for numerous subsequent models to quantify synergistic interactions. It also has led to the widely used combination index (Loewe, 1953), which is simply the left side of Equation (7). If the combination index is less than 1, synergy is said to occur, greater than 1, antagonism. Additionally, this model can be extended to more than 2 compounds (Berenbaum, 1977; Goldoni and Johansson, 2007), where the equation becomes:

(8) $$\begin{array}{r} \frac{a\;}{A}+\;\frac{b\;}{B}\;+\;\frac{c\;}{C}\;\ldots \;\frac{n\;}{N}\;=\;1 \end{array}$$

As with all models, there are limitations and assumptions made. One previously stated assumption is a constant potency ratio for the two dose response curves. As previously stated, curves with constant relative potency will have parallel log dose response curves (Tallarida, 2012; Geary, 2013; Foucquier and Guedj, 2015). Parallel dose response curves and constant potency ratios are considered by some to rarely be the case or to be more of an exception (Loewe, 1953; Geary, 2013). In fact, according to Geary, miniscule deviations from a constant potent ratio could result in a nonparallel log dose effect curves (Geary, 2013). However, Grabovsky and Tallarida (2004) derived similar formulas for nonparallel log dose response curves to deal with such situations, though they may not be as simple as the general equation for constant potency ratios. Furthermore, Loewe Additivity also requires that the dose response curves be accurately estimated individually for each drug in the combination (Foucquier and Guedj, 2015). This is often not a trivial task. A further consideration when using Loewe Additivity is an indeterminate solution resulting, when the result from the conversion of dose A on curve Alpha to a new dose on curve Beta does not align with the conversion of dose B on curve Beta to a dose on curve Alpha (Geary, 2013). According to Geary, this situation occurs frequently (Geary, 2013) and can be illustrated more in-depth by him as well as Tallarida (Tallarida, 2006, 2007; Geary, 2013). Despite these limitations, Loewe Additivity has still been one of the major reference models used and the foundation for many synergy methods.

### Bliss independence

A commonly used alternative to Loewe Additivity is the Bliss Independence model. The Bliss model is based on the idea of noninteraction, that each drug is acting independently of one another (Greco et al., 1995). However, this does not mean that they do not both potentially contribute to the overall effect, but presumably that they take different routes to achieve said effect. An example originally described by Bliss involves an organism that dies from the effects of two distinct compounds. Under the idea of Bliss Independence, these compounds do not interact and perhaps affect different vital systems within the organism. Both compounds do affect the organism, however (Bliss, 1939). The general form of the equation describing Bliss Independence is simply the product of the two fractional responses (Greco et al., 1995):

(9) $$\begin{array}{r} F_{uc}=f_{u1}\;\times \;f_{u2} \end{array}$$

Where *F*_*uc*_ is the fraction unaffected by some outcome for the combination of drugs 1 and 2, *f*_*u*1_ is the fraction unaffected for drug 1 and *f*_*u*2_ is the fraction unaffected for drug two. This can also be written in terms of the fraction affected by some event, as shown originally by Bliss (1939):

(10) $$\begin{array}{r} F_{ac}=f_{a1}+f_{a2}-f_{a1}\;\times \;f_{a2} \end{array}$$

Where *F*_*ac*_ is the fraction affected by some outcome for the combination of drugs 1 and 2, *f*_*a*1_ is the fraction affected for drug 1 and *f*_*a*2_ is the fraction affected for drug two. The two equations can be related by the following:

$$\begin{array}{l} F_{a}\;=\;\left(1-\;F_{u}\right)\to \;\left(1-F_{ac}\right)=\;\left(1-\;f_{a1}\right)\;\times \left(1-\;f_{a2}\right)\\ \;\to \;1-F_{ac}=\;1-\;f_{a1}-\;f_{a2}+\;f_{a1}\;\times \;f_{a2}\;\to \;F_{ac}\\ =\;f_{a1}+\;f_{a2}-\;f_{a1}\;\times \;f_{a2}\text{ Relation }(1) \end{array}$$

A more mathematical interpretation of this can be seen from understanding the idea of probabilistic independence (Foucquier and Guedj, 2015). It is often appropriate to consider this problem in terms of probabilities because responses are often measured as fractions of living or killed components, for example fraction of cell death when administering anticancer drugs. Given this, consider two compounds (drugs, chemicals, etc.), A and B, that act independently of each other. From probability theory,

(11) $$\begin{array}{r} P\left(A+B\right)=P\left(A\right)+P\left(B\right)-P\left(AB\right) \end{array}$$

And, if A and B are independent events, which is assumed under Bliss Independence,

(12) $$\begin{array}{r} P\left(AB\right)=P\left(A\right)\;\times \;P\left(B\right) \end{array}$$

Thus, combining Equations (11) and (12), the common formula for Bliss Independence can be derived,

(13) $$\begin{array}{r} E_{c}=E_{a}+E_{b}-E_{a}\;\times \;E_{b} \end{array}$$

Where *E*_*c*_ is the effect produced by the combination of compounds A and B, at doses a and b, *E*_*a*_ is the effect of compound A at dose a and *E*_*b*_ is the effect of compound B at dose b. The above formula is often used as the reference for how a combination of compounds should act if no synergy or antagonism exists. If the combined effect is greater than what would be expected, as predicted from this formula, synergy is declared, antagonism otherwise. Goldoni shows that this model can be expanded to numerous compounds, though the mathematics become increasingly complex upon using more than 3 compounds (Goldoni and Johansson, 2007).

Though still commonly used as a basic reference model, there has been much criticism over the validity of the Bliss Independence model. The main assumption of this model is that two drugs are acting independently. However, as asserted by Gessner (1974, 1988), for a large proportion of drug interactions, this may not truly be the case. Additionally, for this model to hold true, it must be applicable along the entire dose response curve, something that may not be true in many cases (Gessner, 1988). Advocates of the Loewe Additivity reference model often bring up an additional limitation of the Bliss Independence model. That is that it fails when applied to the “sham mixture” scenario, the basis for the Loewe Additivity model (Berenbaum, 1981; Greco et al., 1995). Many consider the “sham mixture” scenario to be fairly intuitive, so failure to adhere to this could pose a problem when advocating for use of this method.

## Methods for explicit detection of synergy

Aside from simply using the reference models as a baseline for additivity and deviations from that as a measure of synergy, specific methods have been developed for enhanced detection of synergy. Here we discuss some of the more common methodologies along with their benefits and disadvantages. We then further discuss more recent, statistical approaches in a similar manner.

### Isobologram method

**Figure 3:**
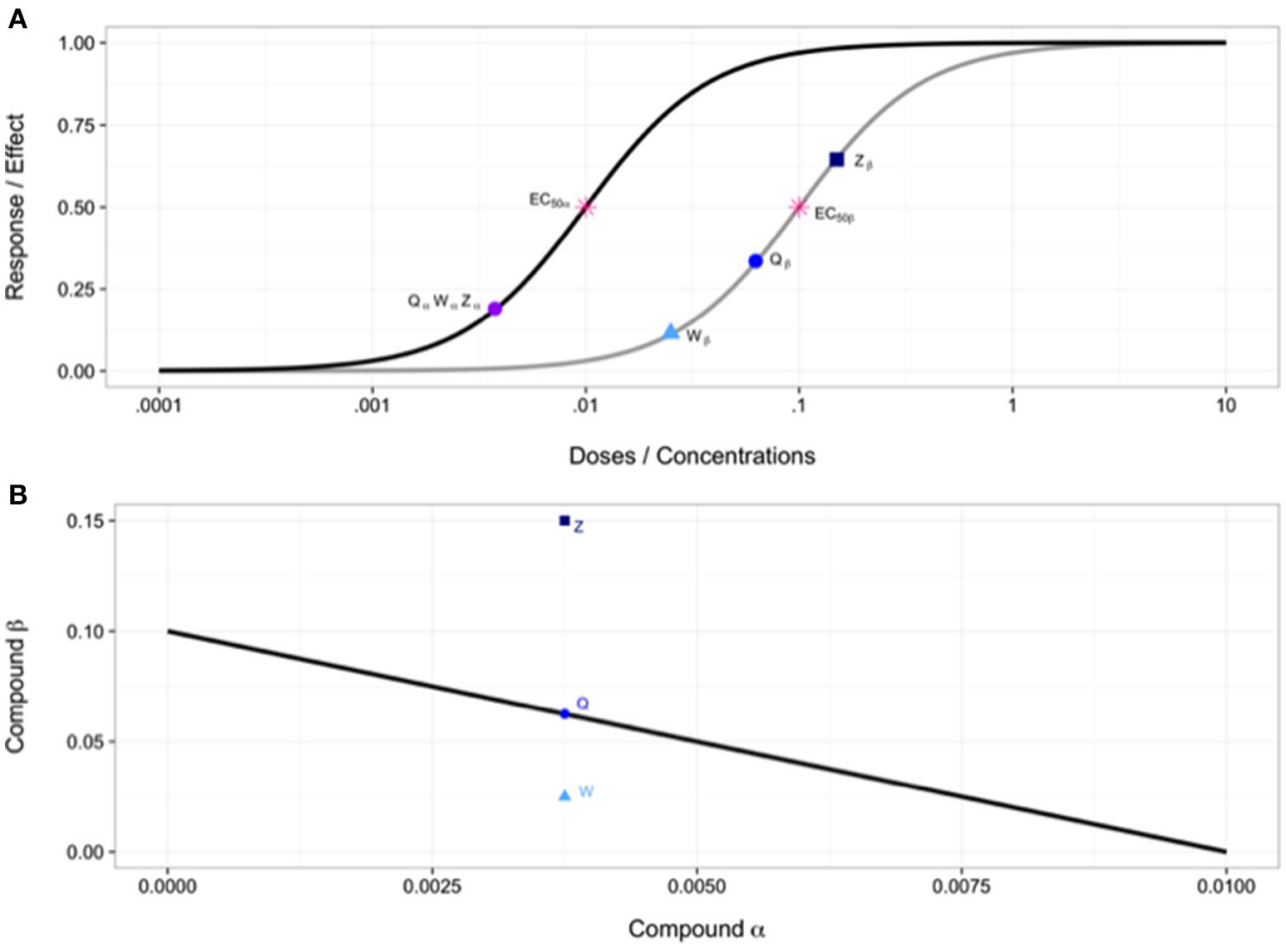
**(A)** Individual dose-response curves with EC_50_ values and various points used in combination in **(B)**. **(B)** Isobologram showing line of additivity. Point W is a combination using doses of W_α_ and W_β_ from **(A)**, similarly for points Q and Z. Point W indicates synergy, point Q indicates additivity and point Z indicates antagonism.

One of the most prolific methods to come out of the Loewe Additivity reference model is the graphical procedure known as the isobologram method. This type of analysis dates back to the late 1800s with Fraser (Fraser, 1870–1871, 1872) and was continued by Loewe (Loewe, 1926; Greco et al., 1995) but was officially published by Loewe (Loewe, 1953, 1957; Tallarida, 2001). An isobologram is a graphical procedure in which doses of one compound are displayed along one axis and doses of a second compound are displayed along the second axis. The entire plot contains combinations of doses for a specific effect level. This method relates to Loewe Additivity because Equation (5) is used to plot a line of additivity, the isobole (Greco et al., 1995). Dose combinations plotted below this line require a lower dose than expected from the Loewe Additivity line and thus can be classified as synergistic, while those plotted above it are antagonistic. This can be seen in Figure 3.

This method is very simple to achieve and a graphical, intuitive interpretation of synergy. However, there have been various drawbacks, further detailed in reviews by Greco et al. (1995) and from a more mathematically perspective by Geary (2013). We will briefly summarize these limitations here. One major drawback is that the approach is too simple for a majority of real world applications, as linear isoboles are relatively rare according to Loewe (1953) and Geary (2013). When dose response curves are nonparallel, as discussed under Loewe Additivity, a nonlinear isobole will result, referred to as a curvilinear isobole (Grabovsky and Tallarida, 2004; Geary, 2013). Another major drawback is that this method lacks a formal statistical framework and does not allow for formally quantifying the intensity of a synergistic interaction (Greco et al., 1995). A similar, relatively popular (Greco et al., 1996) method by Steel and Peckham (1979) was developed to take into account a region around the line of additivity, known as an envelope of additivity. This helps alleviate the problem of simple departures from the additivity line and while there are some other benefits to this method, it also has some of its own drawbacks, discussed more by Greco et al. (1995).

### Chou-talalay method

The Chou-Talalay method is one of the most widely used methods for detecting and quantifying synergistic interactions between two or more drugs (Greco et al., 1996; Boik et al., 2008), having been cited over 7,000 times over the past few decades (Chou and Talalay, 1984; Chou, 2006, 2010). According to Chou, the main equation forming the basis of this method was derived as a unified theory of the Michaelis-Menten, Hill, Henderson-Hasselbalch, and Scatchard equations (Chou and Talalay, 1984; Chou, 2010). They termed this equation the median-effects equation:

(14) $$\begin{array}{r} \frac{f_{a}}{f_{u}}={\left(\frac{D}{D_{m}}\right)}^{m}\;\left(\text{Median-effects equation}\right) \end{array}$$

where *f*_*a*_ is the fraction of cells affected (i.e., killed), *f*_*u*_ is the fraction of cells unaffected (i.e., living), *D* is the dose of drug given, *D*_*m*_ is the median-effect dose and *m* is the sigmoidicity of the dose-effect curve. This equation can be simplified into the following linearized version:

(15) $$\begin{array}{l} log\left(\frac{f_{a}}{f_{u}}\right)=m\;\times \;log\left(D\right)-m\;\times \;log\left(D_{m}\right)\\ \;(\text{Log-linearized median-effects equation}) \end{array}$$

A linear regression can then be applied for the various doses (*D*) and responses (*f*_*a*_/*f*_*u*_). From this, values for *D*_*m*_ and *m* can be estimated. These values can then be used to calculate estimates for variables in the following equation giving the (generalized) combination index (*CI*):

(16) $$\begin{array}{r} CI=\frac{D_{1}}{E_{1}}+\frac{D_{2}}{E_{2}} \end{array}$$

where *D*_1_ and *D*_2_ are the actual drug doses used in the combinations during dosing experiments and *E*_1_ and *E*_2_ are theoretical individual drug levels that would be expected to be needed to achieve the experimentally measured response. While *D*_1_ and *D*_2_ are known from experimental design, *E*_1_ and *E*_2_ can be calculated using the *D*_*m*_ and *m* values previously computed. A *CI* value less than 1 indicates synergism, greater than 1 indicates antagonism, and equal to 1 indicating additivity. This equation can get more complicated depending on the factors such as the exclusivity/independence of the compounds and can be expanded to more than two compounds (Chou and Talalay, 1984).

Though one of the most prolific methods (Greco et al., 1996), there are some considerations to note about this method. Among a number of limitations mentioned by Geary (2013), this method requires drugs have a constant potency ratio (Geary, 2013). Chou has asserted that a constant ratio is not strictly necessary, however a constant potency ratio does result in better accuracy (Chou, 2010). Additionally, in his review, Greco notes three major flaws with the Chou-Talalay Method (Greco et al., 1995). First, he notes calculation of *D*_*m*_ and m, shown in Equation (5), from a median effects plot for mutually nonexclusive drugs can never be correct as dose response curves are primarily nonlinear. Second, Greco shows that the form of one of the equations, for a specific set of parameters, used to calculate the combination index for the mutually nonexclusive case is slightly incorrect. Finally, he mentions that when the median effects plot for the mutually exclusive with interaction case is nonlinear, incorrect conclusions can occur which was shown by Greco as the combination index for part of his data set incorrectly resulting in antagonism instead of synergy due to “incorrect linear extrapolation of the nonlinear median effects curve for the drug combination” (Greco et al., 1995). Even with these considerations, the Chou-Talalay method is by far the most impactful approach to quantifying synergy.

### Response surface

**Figure 4:**
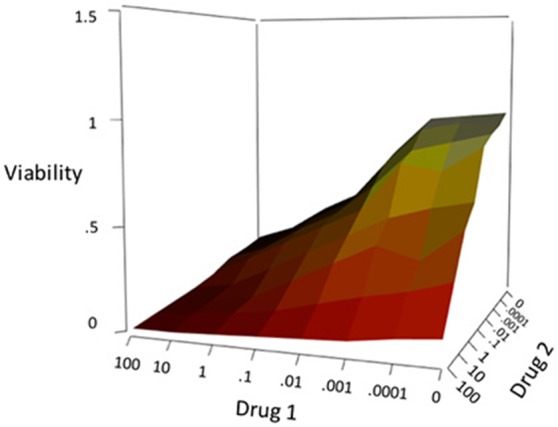
**Example response surface showing viability for the combination of two drugs**.

As an alternative to the reductionist approaches based on curve fits discussed above, response surface modeling tries to use higher dimension data to quantify synergistic responses. The response surface approach has been considered by numerous theorists, each developing a method with a slightly different perspective. The general idea of these types of approaches are to create a 3-dimensional surface consisting of predicted (noninteraction) data points from doses of two drugs. The x- and y-axes are labeled with doses of each drug, similar to an isobologram, but the z-axis is the effect or response at a given combination of doses. The experimentally determined responses for various combinations can be then plotted on this 3D surface where points below or above the surface indicate synergy or antagonism, depending on whether the z-axis is a measurement of inhibition, effect, etc. (Greco et al., 1995). Predictions can be calculated using either Loewe Additivity models, as in the method of Berenbaum (1985), or using Bliss Independence and the Fraction Product Method of Webb (1963) as described by Greco et al. (1995). Similar methods, such as the Method of Prichard and Shipman (1990), also graph response surfaces for the raw data, as opposed to simply plotting points on the predicted surface. Subtraction of the predicted surface from the raw surface can then be performed to get a new 3D surface where peaks above zero and valleys below zero indicate synergy or antagonism, again, depending on the measurement of the z-axis (Greco et al., 1995). Figure 4 shows an example of this approach. A further, more in-depth, discussion of response surface approaches is available in Greco et al. (1995).

### Statistical based approaches

The methods discussed above have a long history of evaluation and use, but methods development is an active field, and a number of new approaches have recently been developed (Boik et al., 2008; Hennessey et al., 2010). Here we discuss two more recent approaches that address some of the limitations of the earlier methods. These approaches bring a statistical framework to assessing synergy, with both frequentist and Bayesian perspectives.

The first method developed by Boik et al. (2008), named MixLow (Mixed-effects Loewe), uses Loewe Additivity as the reference model. The method consists of three basic parts: estimation of dose response curve parameters, calculation of the Loewe Additivity index (i.e., combination index), and calculation of confidence intervals for the index (Boik, 2010). The method assumes a sigmoidal dose response curve and a nonlinear mixed-effects model is used to estimate curve parameters and then uses those parameters to estimate their Loewe Additivity Index and confidence intervals. This method has been favorably compared to the Chou-Talalay Method in simulation experiments (Boik et al., 2008). The major benefits of the MixLow method is that it avoids the necessary preprocessing of the Chou-Talalay method as well as avoiding the need for log-linearization (Boik, 2010). Limitations of this method may be assumptions of a sigmoidal dose response curve and the necessity for fixed-ratio drug combinations.

The second recent method is based on a Bayesian statistical framework and was developed by Hennessey et al. (2010). The idea of analyzing this problem in a Bayesian framework is intriguing and seems to have been overlooked within the field for the most part. This method involves a Bayesian hierarchical nonlinear regression model for the dose response curves of individual and combined agents. Their 3 stage model accounts for “variability between experiments, variability within experiments, and variability in the observed responses of controls.” A priori knowledge, for instance EC_50_ values of a particular agent, can also be incorporated into the model. Markov Chain Monte Carlo is used to estimate the posterior distributions of necessary parameters to calculate what they refer to as the Bayesian Effect Interaction Index. A posterior probability is then calculated and synergy can be declared in a more statistical sense, using probabilities to compare the observed interactions to the expected additive effects. Limitations to this method could include its potential complexity for those unfamiliar with Bayesian statistics and, like many other approaches, that it assumes a constant potency ratio.

## Synergy across disciplines

Thus far, the methods discussed have been developed for the detecting synergy in the biomedical, pharmacological domain. This is likely due to the importance of synergy in the search for more therapeutic treatments for cancer and disease (Greco et al., 1995). There has also been simultaneous development and application of methods for quantifying synergy in other fields, especially in toxicology. Below, we discuss the history of methods development and use in other fields, with a focus on methods developed for domain-specific issues. Although not an exhaustive list by any means, it will hopefully lend a better understanding of different applications of the general idea of synergy.

### Toxicology

The idea of synergy is familiar to the field of toxicology and very related to the traditional biomedical definitions previously discussed in this review. In fact, Bliss originally published his famous reference model, Bliss Independence, in a publication titled “The Toxicity of Applied Poisons” (Bliss, 1939). And, according to Geary, Bliss Independence is often used within the realm of toxicology (Geary, 2013). In their review of mathematical methods in the toxicological realm, Goldoni and Johansson note that both Loewe Additivity and Bliss Independence are used, the former in a more general sense and the latter in more specific subfields such as irradiation (Greco et al., 1995; Groten et al., 2001; Goldoni and Johansson, 2007). The Loewe Additivity model, referred to as Concentration Addition, was used in a comprehensive review article of mixture toxicity within environmental toxicology due to the fact that it is often “recommended for risk assessment purposes” (Cedergreen, 2014). A seemingly common trend among various articles (Hertzberg and MacDonell, 2002; Goldoni and Johansson, 2007; Laetz et al., 2009; Cedergreen, 2014) is to simply look for departures from the reference model being used. Problems with analyses similar to this idea have been discussed by (Greco et al., 1995).

There has been some traction among methods development within the realm of toxicology. One of the older methods for assessing interaction effects and potential synergy was developed by Marking (1977). Mumtaz and Durkin (1992) also developed a method that extends the hazard index. The hazard index was originally introduced by the US EPA to assess the hazard of chemical mixtures (Groten et al., 2001) by focusing on chemicals affecting the same target. The hazard index is limited to assessing mixtures with compounds that produce the same toxic effect through the same mode of action (Groten, 2000). The Mumatz and Durkin method extends the hazard index method to take potential interactions into account using a weight of evidence classification in which information about the individual compounds, such as route of exposure or demonstration of toxicity, is used to more precisely predict the joint action of the compounds in the mixture (Mumtaz and Durkin, 1992; Groten, 2000).

A major problem in the current synergy methodology is the ability to detect synergy among mixtures of more than 2 agents as well as detecting which agents in a multi-agent mixture are actually contributing to the synergistic interactions. For pharmacological applications, the number of drugs/chemicals included in mixtures is typically small, but in environmental mixtures, the mixtures can be complex, and not readily measured. However, mixtures represent more realistic exposure scenarios than the single-agents typically applied in standard studies (Scher, 2012). The basic reference methods, as well as some quantification methods such as, for example, the Chou-Talalay method, can be expanded to more than 2 agents (Chou and Talalay, 1984). However, it is much more difficult to identify the agents within the mixture that do contribute to the synergistic effect without proper, often nearly impossible, experimental design (Foucquier and Guedj, 2015). This has been known to be a problem among drug mixtures and biomedicine but also presents a problem within the field of toxicology.

### Epidemiology

Synergy has also been observed in epidemiological studies as well, often through the observation of diseases that seem to increase the risk of acquiring other diseases. This can be seen in the study of HIV and sexually transmitted diseases (STDs). Coined “epidemiological synergy” by Wasserheit (1992), it was postulated that HIV could augment or even prolong the infectiousness of STDs in a co-infected individual (Wasserheit, 1992). Synergistic interactions are also discussed among risk factors for leukemia in patients that had family histories of breast cancer (Olshan et al., 2001). Schwartz elaborates on the idea of synergy in epidemiology and genetic interactions, describing it as “deviations from additivity” (Schwartz, 2006), the same concept as the synergy defined in the biomedical field. She details an interaction contrast, essentially a specific linear combination of the various independent variable groups within the specific model, as a measure of synergy in this field, also used in subsequent publications (Li et al., 2005; Shen et al., 2005).

### Food sciences

Synergistic interactions have also been observed in food sciences and nutrition. Liu notes that phytochemicals in different foods, that help with cancer prevention, have potential synergistic interactions and that a complex mixture of them is present in whole foods (Liu, 2004). Phytochemicals in food were also implicated in similar analyses by Jacobs and Steffen (2003). The parallels between these and mixtures described in the toxicological world are clear. The chemicals analyzed in studies dealing with phytochemicals, however, are generally looking for helpful effects as opposed to minimizing deleterious ones as many toxicological experiments hope to achieve. Additionally, Gutierrez et al have shown that combinations of plant essential oils and their interactions with food ingredients shows potential synergy, additivity or antagonism using fractional inhibitory concentration indices (Gutierrez et al., 2008, 2009), a similar concept to Loewe Additivity.

## Conclusion

Although the problem of synergy detection and compound interaction is not straightforward, it is of critical importance across multiple academic disciplines, and especially important in cancer treatment. In the current review, we detail the terminology used in quantifying synergy in different disciplines. It can be seen that across disciplines, different nomenclatures have developed and continue to be used today. By unifying these terms in this review, we hope to enhance communication and understanding across disciplines. We also discussed reference methods used to predict the effect of individual drugs under an additive model, as well as benefits and disadvantages to the two most common additive models used. We then detail the methods most commonly used for quantifying synergy, and highlight emerging methods. Finally, we discuss applications of synergy quantification in a range of fields.

Although the pace of methods development has steadily increased since the early twentieth century, there remain challenges with the current, common methodologies for measuring synergy. The majority of currently used methods discussed lack much statistical rigor (Greco et al., 1995; Foucquier and Guedj, 2015), as demonstrated by Greco et al in their 1995 review paper. Additionally, many of the discussed methods assume a constant potency ratio which is rarely biochemically appropriate. And while some methods have standalone software, the Chou-Talalay method, or an R package, MixLow, many still require user implementation or knowledge of particular programming languages to run supplied pieces of code as opposed to having a dedicated tool for running the method. Synergy is something that merits our attention and pursuit (Geary, 2013), and, given its potential importance across many domains, development of new methods and enhancements to existing methods could have far-reaching impacts.

Before concluding, we would also like to highlight one area of importance that is often unmentioned in the current methodological literature: synergistic interactions in sequential therapeutic approaches. This occurs when one or more drugs is administered prior to another drug or mixture of drugs and a synergistic interaction occurs between the first and second administration of drugs. This has been shown to exist among anti-cancer drugs as well as between epigenetic drugs and anti-cancer drugs (Azrak et al., 2004; Vijayaraghavalu et al., 2013), for example. Quantifying this type of synergistic interaction presents its own unique challenges. Challenges such as determining which drugs are interacting if working with mixtures or the significance of specific timing between administration of the drugs needs to be considered. There is an emerging clear gap between the known clinical utility of this type of dosing strategy, and quantitative methods to characterize this that needs to be addressed in future studies.

## Author contributions

All authors listed, have made substantial, direct and intellectual contribution to the work, and approved it for publication.

## Funding

This work was supported by NIH NCI RO1CA161608 and NIH P01 CA142538 from the National Cancer Institute.

### Conflict of interest statement

The authors declare that the research was conducted in the absence of any commercial or financial relationships that could be construed as a potential conflict of interest.
